# Molecular and Cellular Mechanisms of Inflammation and Tissue Regeneration

**DOI:** 10.3390/biomedicines11051416

**Published:** 2023-05-10

**Authors:** Anne-Laure Favier, Krisztina Nikovics

**Affiliations:** Imagery Unit, Department of Platforms and Technology Research, French Armed Forces Biomedical Research Institute, 91223 Bretigny sur Orge, France; anne-laure.favier@intradef.gouv.fr

Over the past 70 years, significant progress has been made in understanding the molecular and cellular mechanisms of inflammation and tissue regeneration. This has become most evident in the last 20 years when revolutionary techniques in biology have exponentially increased the number of publications in the field. Following tissue damage caused by infection, mechanical or toxic injury, or autoimmune diseases, the healing process involves a series of highly regulated molecular and cellular processes that lead to the restoration of tissue homeostasis. If any step in the regeneration process is dysfunctional, chronic inflammation, tissue fibrosis or tumor formation can develop [1].

There are three overlapping steps in wound healing: (1) coagulation and inflammation, then (2) the proliferation and formation of new tissue, and (3) finally the tissue remodeling [2]. The initial phase of acute wound healing is the coagulation and the formation of a temporary wound matrix. This phase begins immediately after the injury and is completed within a few hours [3]. Inflammation is crucial to the clean-up-repair process. Early inhibition of inflammation can hinder regeneration processes [4]. Inflammation is associated with the activation of the innate immune system. At the site of inflammation, neutrophils appear first, followed by monocytes, which may differentiate into macrophages. The main function of macrophages and immune cells is to remove cell debris and microorganisms. These cells, in addition to the functions mentioned below, play an essential role in preparing the next phase by coordinating cellular processes [5]. The second phase starts with the division of the cells. This process allows damaged and lost structures to be replaced. Granulation tissue formed by the extracellular matrix (ECM) and new blood vessels generated by angiogenesis fills the lesion. This process takes 2–10 days [6]. In the final phase, the blood vessels regress, the inflammation resolves, and the granulation tissue become functional tissue. In this phase, the ECM transforms from a temporary ECM to a permanent collagen matrix. This phase starts 2–3 weeks after injury and can last up to years if tissue regeneration is inadequate [7].

The role of immune cells, in particular macrophages, in the inflammatory process has long been known, but it has recently been discovered that these immune cells also play an important role in the second and third stages of tissue regeneration [8,9,10,11]. They are involved in the activation of stem/progenitor cells, the clearance of damaged tissues, the remodeling of the extracellular matrix, and the promotion of angiogenesis [12]. Macrophages and molecular actors of tissue regeneration continuously produce soluble mediators and extracellular vesicles that stimulate fibroblasts, immune cells, and stem/progenitor cells [12,13,14]. Soluble mediators, together with extracellular vesicles (which may contain various bioactive molecules such as nucleic acids, proteins, lipids, and sugars), play an important role in cell-to-cell communication at all steps of regeneration (Figure 1) [15,16].

Several interesting findings and overviews were shortly mentioned to illustrate molecular and cellular actors involved in inflammation and tissue regeneration.

Rangarajan and co-workers (2022) reviewed the effects of different pro-resolving mediators on atherosclerosis [17]. Atherosclerosis is a chronic inflammation that can last for several years. The healing of this disease can be greatly facilitated by the presence of various pro-resolving mediators such as resolvin, lipoxin, maresin, and protectin. The study of the complex temporal and functional relationships of these molecules offers a new approach to the treatment of tissue regeneration.

Durand and co-authors (2022) investigated the osteogenic effects of two different biomaterials (metakaolin and polymethylmethacrylate) in the Masquelet-induced membrane in a rat model animal [18]. They showed that the nature of the biomaterials influences the immune microenvironment and macrophage responses, which strongly affects bone regeneration. The more intense osteogenic effect of metakaolin was attributed to a higher number of M1- and M2-like macrophages and to a more intense expression of transforming growth factor-β (TGF-β) and bone morphogenetic protein-2 (BMP-2).

Spinal cord injury often causes paralysi and currently no therapy is available. Following injury, nerve and glial cells die as a result of inflammation. In addition, activation of microglial cells and infiltration of macrophages and lymphocyte cells can be observed in the injured tissue. Wu and colleagues (2022) showed that the bile acid tauroursodeoxycholic acid (TUDCA) was able to inhibit inflammation and promote the temporary recovery of motor function in rats [19]. Unfortunately, these positive effects are not noticeable in the long term, so the authors do not recommend TUDCA for the treatment of spinal cord injury.

Toll-like receptor 8 (TLR8), localizes in the endosome, is able to bind to single-stranded RNAs of viral and bacterial origin. In human monocytes and macrophages, pro-inflammatory cytokines and type I interferons are secreted in response to the binding. Nilsen and colleagues (2022) investigated this mechanism [20]. Their work showed that the Toll-interleukin 1 receptor domain adaptor protein (TIRAP) played an important role in regulation and was involved in signal transduction as an adaptor protein. This protein promotes the translocation of a transcription factor, interferon regulatory factor 5 (IRF5), into the nucleus and the production of IRF5-dependent cytokines.

Alumina nanoparticles (Al_2_O_3_ NPs) are one of the most frequently produced particles in the world. Dekali and colleagues (2022) reviewed the current knowledge on the effects of Al_2_O_3_ NPs on animal health [21]. Al_2_O_3_ NPs have been shown to significantly increase the risk of inflammation, pulmonary fibrosis, reduced lung function, and increased risk of lung cancer in the lung parenchyma of various animals. In contrast, in vitro studies on the effects of Al_2_O_3_ NPs on lung cells have been rather controversial.

Recently, there has been renewed interest in analyzing the mechanism of action of chemical warfare agents derived from organophosphorus materials. François and colleagues (2022) analyzed the long-term effects of 4′-nitrophenyl isopropyl methyl phosphonate (NIMP), a sarin surrogate [22]. The experimental mice received two sub-lethal doses of NIMP and were studied for 6 months. Six months after exposure, inflammation of the gut, anxiety-like behavior, and significant changes in leukocyte count was observed.

The development of inflammatory and tissue regeneration disorders is largely due to an imbalance between reactive oxygen species (ROS) and endogenous antioxidants. Perez and colleagues (2022) reviewed the relationship between the effects of antioxidants on neuronal remyelination and muscle regeneration [23]. In summary, it was pointed out that if the remyelination of damaged nerve cells is inadequate, the regeneration of muscle cells will not be efficient. In addition, antioxidants have been shown to facilitate the remyelination of nerve cells, which promotes muscle cell regeneration.

Hart and Nakamura (2022) reviewed the most important cell therapy methods for the regeneration of damaged musculoskeletal tissue [24]. The cell therapies that have been used over the past 30 years and have greatly improved the regeneration of musculoskeletal tissues have been Platelet-rich Plasma (PRP) or mesenchymal stem cell (MSC) therapies. Factors in the PRP or secreted by the MSC activate the regenerative capacity of endogenous cells. To ensure optimal regeneration, the first step is to reduce inflammation and then introduce properly activated cells into the damaged tissue. Cell therapy was less effective when the cells were injected into the bloodstream, as the cells were probably not able to reach the site of injury.

Failure of tissue regeneration can have serious clinical consequences, such as tumor formation because proteins and cells involved in signaling mechanisms that are important for healing are also involved in cancer metastasis. In their review, Lopez and colleagues (2022) compared the relationship between wound healing and metastasis [2]. They have underlined the role of oxidative stress in these processes. It is very important to reduce oxidative stress, for example by limiting its production, using scavenger agents, and increasing the antioxidant capacity of the cells. These treatments offer interesting therapeutic options that can promote proper tissue regeneration and prevent metastasis.

In our laboratory, we have investigated a method to identify mRNA transcripts in cryosections of undecalcified rat bone. In collaboration with the Institute of Plant Sciences of Paris-Saclay (IPS2), in situ hybridization and hybridization chain reaction (in situ-HCR) was adaptedto better understand gene expression in in situ bone tissue [25]. Our objective was to study a section of a whole rat femur. The muscle significantly delayed the penetration of the decalcifying solution (EDTA) into the bone, so paraffin embedding was not applicable. We have developed an improved version of the CryoJane tape transfer system for making bone tissue sections. This technique was used to investigate the expression of different genes in bone tissue by in situ-HCR.

As the phenotype of in vivo macrophages is still poorly understood, we used the above techniques to identify in vivo macrophages in regenerating bone [26]. There is no cell surface marker available to identify macrophage subtypes that can distinguish between different subtypes. Another approach to determining macrophage subtypes is to identified the patern of cytokines they express. However, since cytokines are usually secreted, immunostaining techniques are not suitable for detecting subtypes. We have shown that in situ-HCR hybridization is one of the most suitable methods to detect cytokines in order to characterize macrophage subtypes. This technique is based on the detection of messenger RNA (mRNA) of targeted genes.

Understanding the mechanisms of inflammation and tissue regeneration is of great scientific and clinical importance. However, the molecular and cellular mechanisms through which they exert their effects are still largely unknown. The study of these processes is essential for the development of therapeutic strategies aimed at tissue repair. We hope that readers of this special issue of Biomedicines will enjoy reading the excellent papers of many of the leading scientists in the field. We hope also that future generations of researchers will be inspired by the topics published in this special issue to further improve our knowledges in this field.

## Figures and Tables

**Figure 1:**
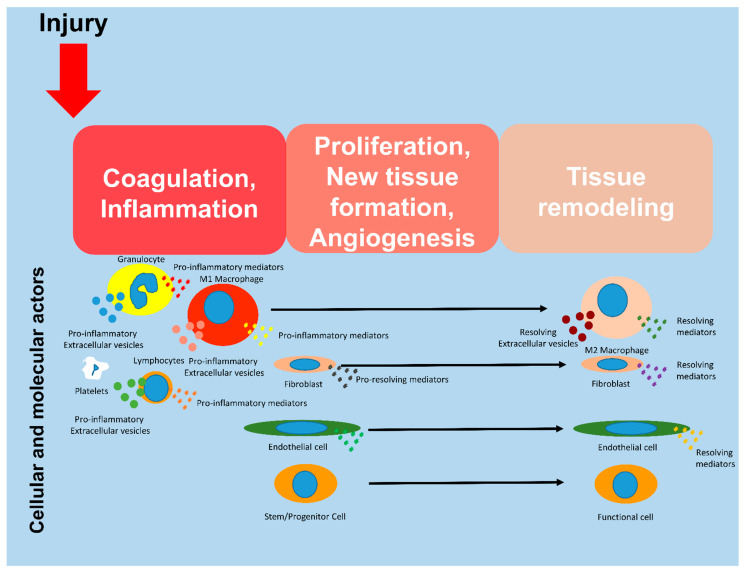
Molecular and cellular actors during tissue regeneration. Regeneration occurs after three schematical steps: coagulation/inflammation, profiferation/angiogenesis and finally tissue remodeling.
